# Gradual *in vitro* Evolution of Cefepime Resistance in an ST131 *Escherichia coli* Strain Expressing a Plasmid-Encoded CMY-2 β-Lactamase

**DOI:** 10.3389/fmicb.2019.01311

**Published:** 2019-06-12

**Authors:** Valentina Donà, Maximilian Scheidegger, João Pires, Hansjakob Furrer, Andrew Atkinson, Baharak Babouee Flury

**Affiliations:** ^1^Institute for Infectious Diseases, University of Bern, Bern, Switzerland; ^2^Graduate School for Cellular and Biomedical Sciences, University of Bern, Bern, Switzerland; ^3^Department of Infectious Diseases, Bern University Hospital, University of Bern, Bern, Switzerland

**Keywords:** CMY-2, CMY-69, WGS, resistance evolution, ST131, cefepime

## Abstract

**Background:**

In a previous report, a clinical ST131 *Escherichia coli* isolate (*Ec*-1),producing a plasmid-encoded AmpC β-lactamase CMY-2, evolved *in vivo* under cefepime (FEP) treatment to the FEP-resistant *Ec*-2 strain expressing an extended-spectrum β-lactamase CMY-33. To compare factors responsible for *in vitro* and *in vivo* FEP resistance, we reproduced *in vitro* FEP resistance evolution in *Ec*-1.

**Methods:**

FEP-resistant mutants were generated by subjecting *Ec-1* (FEP MIC = 0.125 mg/L) to sub-inhibitory concentrations of FEP. MICs were obtained by broth microdilution or *E*test. Strains were sequenced on an Illumina HiSeq platform. Transcriptional levels and plasmid copy numbers were determined by real-time PCR. Outer membrane proteins (OMPs) were extracted and separated by SDS-PAGE. Growth kinetics was evaluated by measuring OD_450_.

**Results:**

The CMY-2 expressed by *Ec*-1 evolved to a CMY-69 (strain EC-4) by an Ala294Pro substitution after 24 passages. After 30 passages, the FEP MIC increased to 256 mg/L (strain EC-32). SDS PAGE did not reveal any lack of OMPs in the mutant strains. However, *bla*_CMY_ transcription levels were up to 14-times higher than in *Ec*-1, which was partially explained by mutations in the upstream region of *repA* resulting in a higher copy number of the *bla*_CMY_-harboring IncI1 plasmid. All mutants showed a slight growth defect but no significant difference in relative growth rates compared to *Ec*-1.

**Conclusion:**

*In vitro* sub-inhibitory concentrations of FEP resulted in the selection of resistance mutations altering the H-10 helix of the CMY-2 and increasing the plasmid copy number. Appropriate dosing strategies may help preventing resistance evolution during treatments.

## Introduction

Cefepime (FEP) consumption in Swiss hospitals is among the highest in Europe (Pluss-Suard et al., 2012). This 4th generation cephalosporin is relatively stable to hydrolysis through chromosomally encoded AmpC β-lactamases, and therefore represents the treatment of choice in AmpC producers that do not harbor extended-spectrum beta-lactamase enzymes (ESBLs) (Harris and Ferguson, 2012; Hilty et al., 2013) or class A or B carbapenemases, which are able to hydrolyze the drug (Endimiani et al., 2008).

CMY-2 is a plasmid-encoded AmpC (pAmpC) β-lactamase deriving from the chromosomally encoded AmpC of *Citrobacter freundii* (Bauernfeind et al., 1996). The *bla*_CMY–2_ gene is often located on large plasmids of the IncI and IncA/C incompatibility groups, which have been identified in *Escherichia coli* and other Enterobacteriaceae worldwide (Philippon et al., 2002; Baudry et al., 2009).

*Escherichia coli* expressing plasmid-mediated CMY-2 are usually still susceptible to FEP. However, in the past, resistance to FEP has sporadically been reported in *E. coli* with de-repressed AmpCs and in strains with compounding porin deficiencies (Mammeri et al., 2007, 2010; Haenni et al., 2014). More recently, FEP-resistant *E. coli* isolates producing extended-spectrum AmpCs (pESACs) have been described, which arose through two to four amino acid deletions in the H-10 helix of the CMY-2 (Doi et al., 2009). Notably, the *in vivo* development of FEP-resistance under FEP treatment through the evolution of a pAmpC CMY-2 to a pESAC CMY-33 has been recently reported in a hyperepidemic sequence type (ST) 131 *E. coli* clone (Pires et al., 2015). In this report, the FEP-susceptible *E. coli* isolate *Ec*-1 evolved to the FEP-resistant *Ec*-2 isolate as a consequence of a 6-bp deletion in the *bla*_CMY–2_ gene harbored on an IncI1 plasmid, which resulted in a Leu293-Ala294 deletion in the H-10 helix that expanded the substrate spectrum of the enzyme. The authors also confirm the absence of any ESBL genes by CT-103 Checkpoint Microarray, which was important to rule out any potential contribution of such β-lactamases to the observed phenotype. In fact, differentiation of strains expressing AmpCs and ESBLs based on their 3rd and 4th generation cephalosporin-resistance can be challenging due to overlaps in the resistance phenotype (Jacoby, 2009). For instance, the presence of ESBLs can be masked by high-level AmpC production (EUCAST, version 6.0, 2016). On the other hand, AmpC-producers typically test positive for the ESBL screening test, but negative in the confirmatory test. However, this phenotype may also be observed in peculiar TEM mutants, strains harboring OXA-type ESBLs or carbapenemases or expressing high levels of *bla*_TEM–1_. This scenario is further complicated by the emergence of strains co-harboring pAmpC and ESBL-genes (Ghosh and Mukherjee, 2016).

In this study, we reproduced *in vitro* the FEP-resistance evolution in the original FEP-susceptible *E. coli* isolate *Ec*-1 by gradually subjecting the strain to increasing sublethal drug concentrations. The objective was to determine the role of the *bla*_CMY_ in the resistance evolution and to identify hotspots for mutations responsible for FEP resistance. We also aimed to compare mechanisms evolved *in vitro* with those observed *in vivo*, and assess the *in vitro* fitness of the resistant mutants.

## Materials and Methods

### Bacterial Strains

The *E. coli* clinical isolates *Ec*-1 expressing *bla*_CMY–2_ and *Ec*-2 expressing *bla*_CMY–33_ used in this work were isolated as part of a previous study (Pires et al., 2015). The porin-deficient mutants *E. coli* MH513 MC4100 *araD*^+^ Φ(*ompF*::*lacZ*) and MH225 MC4100 *malQ7* Φ(*ompC*::*lacZ*) were described in a previous study (Liu and Ferenci, 1998).

### Antimicrobial Susceptibility Testing

The MICs for several antibiotics were determined by broth microdilution in Mueller–Hinton broth (BBL, Becton Dickinson) using the Sensititre GNX2F and ESB1F plates (Trek Diagnostic Systems). MIC values were interpreted in accordance with the 2016 European Committee on Antimicrobial Susceptibility Testing (EUCAST) criteria (version 6.0, 2016), besides for cephalothin, cefoxitin and polymyxin B, for which clinical and laboratory standards institute (CLSI) criteria (M100-S26, 2016) were applied. MICs were also determined in the presence of the efflux pump inhibitor phenyl-arginine-β-naphthylamide (PaβN) (Sigma-Aldrich) at a final concentration of 25 mg/L and 10.4 mg/L (Saw et al., 2016). MICs for FEP were additionally determined by Etest (BioMérieux).

### Serial Passages

Strain *Ec*-1 and its derivatives were passaged in the presence of sub-inhibitory concentrations ($\frac{1}{4}$ of the respective MIC) of FEP. Briefly, one single colony of *Ec*-1 grown ON at 37°C on a cation-adjusted Mueller-Hinton agar plate was picked and grown in 10 ml of cation-adjusted Mueller-Hinton broth at 37°C for 24 h. Then, 50 μl were inoculated into 10 ml cation-adjusted Mueller-Hinton broth containing a FEP concentration at $\frac{1}{4}$ of the initial MIC of the strain and incubated at 37°C for 24 h with shaking at 120 rpm (first passage). Subsequently, 50 ul of the resulting culture were diluted in 10 ml cation-adjusted Mueller-Hinton broth with the same FEP concentration and incubated at 37°C for 24 h for two additional times (second and third passage). After these three successive passages in the subinhibitory broth, 50 ul of the broth were diluted and streaked onto a cation-adjusted Mueller-Hinton agar plate which was incubated ON at 37°C. Three representative colonies were randomly selected from the plate and frozen at −80°C for further analysis. The three selected clones were subjected to MALDI-TOF analysis to exclude contaminations and the FEP MIC (MIC_FEP_) and the *bla*_CMY_ gene type were determined by Etest and PCR/Sanger sequencing, respectively. One clone was then randomly chosen to repeat the passages in subinhibitory broth as described above, with the FEP concentrations for the subsequent three passages increased according to the MIC results, i.e., $\frac{1}{4}$ of the MIC. The same procedure was also performed in parallel without antibiotic pressure (negative controls). Aliquots of the bacterial cultures were regularly sampled and kept at −80°C for further molecular investigations.

### Detection of Sequence Alterations in *bla*_CMY_ and Whole Genome Sequencing

The strains were subjected to Sanger sequencing of the *bla*_CMY_-coding region amplified using the primers listed in Supplementary Table S1. Total DNA was extracted from the strains with the QiaAMP Mini Kit (Qiagen) and used to construct libraries with the Nextera DNA Library Prep Kit (Illumina) following manufacturer’s instructions. The libraries were sequenced on an Illumina HiSeq platform (Illumina) using the 2 × 150 bp paired-end sequencing protocol. Reads were trimmed with Trimmomatic v.0.33 (Bolger et al., 2014) and *de novo* assemblies were subsequently performed with SPAdes v.3.9.0 (Bankevich et al., 2012). STs based on multi-locus sequence typing (MLST), plasmid replicon types and the presence of resistance genes were determined *in silico* on an online platform (Center for Genomic Epidemiology^1^) using MLST 1.8, PlasmidFinder 1.3, and ResFinder 2.1, respectively. For single nucleotide polymorphism (SNP) and indel analysis, read-mappings of the Illumina reads to the assembled reference sequences were performed with BWA-MEM v0.7.13 (Zhu et al., 2013) with default settings and subsequently analyzed with the Geneious software v.10.2.3^2^ (Kearse et al., 2012). Filtering parameters for variant calling were a minimum variant frequency of 0.8 and a maximum variant *P*-value of 10^−6^. The read-mappings were also used to confirm that the mutant strains were deriving from the parental strain.

### Evaluation of Transcription Levels and Plasmid Copy Number

Real-time PCR was used to measure mRNA expression levels of *bla*_CMY_, *ompF*, *ompC* and the relative copy number of IncI1 replicon region with primers listed in Supplementary Table S1. For the determination of changes in mRNA expression levels between the parental and derivative strains of *bla*_CMY_, *ompF* and *ompC*, mid-logarithmic growth cultures (0.5 ml) were taken and treated with RNAprotect reagent (Qiagen). RNA was extracted with RNeasy Mini Kit (Qiagen) and the eluate was treated with DNase I (Qiagen) according to the manufacturer’s instruction. The reverse transcription (RT)-PCR was subsequently performed using the *Power* SYBR^®^Green RNA-to-C_T_ 1-*Step* Kit (Thermo Fisher Scientific) and a QuantStudio 7 Flex Real-Time PCR System (Thermo Fisher Scientific) using an annealing temperature of 60°C. Transcript measurements were carried out in triplicate and relative quantification of target genes was calculated with the 2^−ΔΔCT^ method using *rpoD* as a reference (Sauer et al., 2004; Marcoleta et al., 2016), as described previously (Livak and Schmittgen, 2001), with the original susceptible strain *Ec*-1 used as the calibrator (Supplementary Table S1). To assess a correlation between changes in *bla*_CMY_ transcription and plasmid copy numbers, total DNA from mid-logarithmic growth cultures was extracted using the Qiagen DNeasy Mini Kit (Qiagen) and treated with RNase according to the manufacturer’s instructions. Real-time PCR was performed as described above, using the extracted DNA as template. Data analysis was performed as described above, using the single-copy chromosomal gene *ampD* for normalization.

### Examination of Porin Expression

Outer membrane proteins (OMPs) were extracted according to the rapid procedure of Carlone et al. (1986). Briefly, cells were disrupted with FastPrep homogeniser (MP Biosciences) using glass beads (Sigma-Aldrich) for four cycles of 50 s at 4 m/s. After washing steps with 10 mM HEPES, the membrane pellets were suspended in 50 μl of 10 mM HEPES and stored at −80°C. This procedure routinely yields preparations containing approximately 2 mg of protein per ml. *E. coli.* MH513 and MH225 were used as reference strains for OmpC and OmpF expression, respectively. Protein amounts were measured using Nanodrop (Thermo Fischer Scientific). The proteins were precipitated with acetone and resuspended in 20 μl of solubilization buffer. Fifteen microliter of protein-buffer solution were separated on a 10% Laemmli SDS-PAGE gel (Laemmli, 1970).

### Bacterial Growth Curves

Cultures were grown overnight at 37°C in cation-adjusted Mueller-Hinton broth. Cells were then diluted 1:1000 in fresh cation-adjusted Mueller-Hinton broth before inoculation in quadruplicate on a 96 well plate. The plate was placed inside a tunable microplate reader (Versamax, Molecular Devices), which was programmed with the Softmax Pro software (Molecular Devices) to incubate the plate at 37°C and measure the optical density at 450 nm (OD_450_) every 30 min for 22 h. Three biological replicates were performed for each strain.

To estimate generation times, the exponential phase for each strain was first estimated and confirmed by linear regression of the lnOD_450_ values. Two time points within the exponential phase (ODf and ODi) were then chosen to calculate the generation time, as done previously Lange et al. (2018). Relative growth rates were calculated by dividing the generation time of the mutant strain with that of the parental strain. The obtained relative growth rates were subsequently subjected to a two-tailed *t*-test, in order to identify statistically significant differences in the relative growth rate of the mutant strains and the parental strain *Ec*-1, for which the relative growth rate was set to 1.

### Nucleotide Accession Numbers

The whole-genome shotgun sequences of *Ec*-1 and its derivative resistant strains are deposited in DDBJ/ENA/GenBank under the following accession numbers: *Ec*-1, QEVU00000000; EC-4, QDKR00000000; EC-16, QDKS00000000; EC-32, QDKT00000000; and *Ec*-2, QDKU00000000.

## Results

### *In vitro* FEP Resistance Evolution

*Ec*-1 with an initial MIC_FEP_ of 0.125 mg/L evolved to EC-4 with an intermediate resistance to FEP (MIC_FEP_ = 4 mg/L) after 24 passages in broth containing sub-inhibitory concentrations of FEP. After 27 passages, EC-16, resistant to FEP (MIC_FEP_ = 32 mg/L), and after 30 passages EC-32, highly resistant to FEP (MIC_FEP_ = 256 mg/L), were obtained. The MIC_FEP_ of the *in vivo* evolved mutant strain *Ec*-2 was 48 mg/L. The addition of the efflux pump inhibitor PaβN at concentrations of 10.4 and 25 mg/L did not change the MIC_FEP_ in any of the strains.

The MICs for different antibiotics for *Ec*-1 and the *in vitro* (EC-4, EC-16, and EC-32) and *in vivo* (*Ec*-2) derivative resistant strains are listed in Table 1. Of note, *Ec*-1, *Ec*-2, and EC-4 were susceptible to piperacillin-tazobactam, whereas EC-16 and EC-32 were resistant (MIC > 64 mg/L).

**Table 1:** MICs, sequence type, CMY variant and number of passages with cefepime of *E. coli Ec*-1, and its cefepime-resistant derivatives.

		*Ec*-1	*Ec*-2	EC-4	EC-16	EC-32
Antibiotic MIC, mg/L (interpretation)^a^	Cefepime^c^	0.125 (S)	48 (R)	4 (I)	32 (R)	256 (R)
	Ampicillin	≥32 (R)	≥32 (R)	≥32 (R)	≥32 (R)	≥32 (R)
	Piperacillin-tazobactam	≤4 (S)	8 (S)	8 (S)	64 (R)	64 (R)
	Ticarcillin-clavulanate	≤8 (S)	≥256 (R)	≥256 (R)	≥256 (R)	≥256 (R)
	Cephalothin^b^	≥32 (R)	≥32 (R)	≥32 (R)	≥32 (R)	≥32 (R)
	Cefoxitin^b^	64 (R)	≥128 (R)	≥128 (R)	≥128 (R)	≥128 (R)
	Cefotaxime	4 (R)	64 (R)	64 (R)	≥128 (R)	≥128 (R)
	Cefotaxime-clavulanate	4 (NA)	32 (NA)	32 (NA)	64 (NA)	64 (NA)
	Ceftriaxone	8 (R)	128 (R)	128 (R)	≥256 (R)	≥256 (R)
	Ceftazidime	16 (R)	≥256 (R)	≥256 (R)	≥256 (R)	≥256 (R)
	Ertapenem	≤0.25 (S)	≤0.25 (S)	≤0.25 (S)	≤0.25 (S)	≤0.25 (S)
	Imipenem	≤0.5 (S)	≤0.5 (S)	≤0.5 (S)	≤0.5 (S)	≤0.5 (S)
	Meropenem	≤1 (S)	≤1 (S)	≤1 (S)	≤1 (S)	≤1 (S)
	Ciprofloxacin	≤0.25 (S)	≤0.25 (S)	≤0.25 (S)	≤0.25 (S)	≤0.25 (S)
	Amikacin	≤4 (S)	≤4 (S)	≤4 (S)	≤4 (S)	≤4 (S)
	Gentamicin	≤1 (S)	≤1 (S)	≤1 (S)	≤1 (S)	≤1 (S)
	Colistin	≤0. 25 (S)	≤0. 25 (S)	≤0. 25 (S)	≤0. 25 (S)	≤0. 25 (S)
	Polymyxin B^b^	≤0. 25 (S)	≤0.25 (S)	≤0.25 (S)	≤0. 25 (S)	≤0.25 (S)
	Tigecycline	≤0.25 (S)	≤0.25 (S)	≤0.25 (S)	≤0.25 (S)	≤0.25 (S)
	Aztreonam	4 (I)	≥32 (R)	≥32 (R)	≥32 (R)	≥32 (R)
	Co-trimoxazole	≤0.5 (S)	≤0.5 (S)	≤0.5 (S)	≤0.5 (S)	≤0.5 (S)
Sequence type (ST)^d^		ST131	ST131	ST131	ST131	ST131
CMY variant		2	33	69	69	69
Passages with cefepime (at $\frac{1}{4}$ of MIC)		–	–	24	27	30

*^a^According to the EUCAST criteria (v6.0, 2016). MIC values (besides for cefepime) were obtained implementing the GNX2F, and ESB1F plates (Trek diagnostics). ^b^According to CLSI criteria (M100-S26, 2016). ^c^MIC was obtained using Etest^®^. ^d^The ST type was determined with the MLST 1.8 tool available on the Center for Genomic Epidemiology online platform.*

### Molecular Characterization of the Resistant Mutants

PCR amplification and Sanger sequencing of the *bla*_CMY_-coding region indicated that in EC-4, EC16 and EC-32, the CMY-2 had evolved to a CMY-69 due to the SNP G940C resulting in an Ala294Pro substitution [based on the amino acid numbering scheme of *Enterobacter cloacae* P99 (Galleni et al., 1988)] in the H-10 helix (Supplementary Figure S1). These mutations were subsequently confirmed by whole-genome sequencing (WGS), while no mutations in the promoter region of *bla*_CMY_ were found in any of the resistant strains compared to *Ec*-1. No transposition or duplication of the gene was detected in the assemblies and the absence of any ESBL genes was confirmed by *in silico* analysis in the parental strain and its derivatives.

**FIGURE 1:**
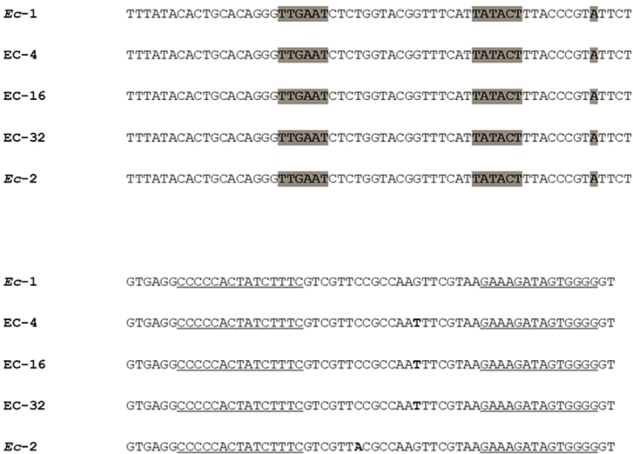
Sequence of the *inc* promoter and hairpin loop region in strain *Ec*-1 and its *in vitro* and *in vivo* mutant derivatives [adapted from Kurpiel and Hanson, 2012] Point mutations are denoted with bold nucleotides. Underlined sequences represent complementary sequence arms of the *inc* RNA stem loop. The transcriptional start site and putative -10 and -35 promoter hexamers for *inc* are highlighted in gray.

SNPs were found within the hairpin loop region of the *inc* gene located on the IncI1 plasmid, consisting of a G24T mutation in the *in vitro* mutants EC-4, EC-16 and EC-32, and a C31A mutation in the *in vivo* evolved mutant *Ec*-2 (Figure 1). Of note, it has been recently shown that mutations in this region cause an increase in plasmid copy number (Kurpiel and Hanson, 2012).

No other point mutations potentially associated with FEP resistance were identified in the *in vitro* resistant mutants. However, the *in vivo* evolved mutant *Ec*-2 harbored two non-synonymous SNPs in the *rpoC* gene resulting in a G125R and a V289A substitution in the subunit beta’ of the DNA-directed RNA polymerase.

### Gene Transcription Levels and Plasmid Copy Number

Since an increase in the expression levels of the *bla*_CMY_ gene or a decrease in the transcription of the porin-encoding genes *ompF* and *ompC* may be involved with FEP resistance, the mRNA levels of these genes were evaluated for all strains. The mutants evolving from *Ec*-1 exhibited 2- to 14-fold increases in *bla*_CMY_ transcription compared to the parental strain *Ec*-1. For the *in vitro* mutants, higher levels of *bla*_CMY_ transcription correlated with higher MIC_FEP_, whereas the clinical mutant strain *Ec*-2 showed the lowest increase in transcription levels (approximately twofold) (Table 2). Since the mutations identified in *inc* may be associated with an increase in plasmid copy number, which could explain the observed increase in *bla*_CMY_ transcription, we quantified and compared the copy number of the IncI1 plasmid of *Ec*-1, and its resistant derivatives. All mutants showed increases in plasmid copy number compared to *Ec*-1, i.e., 2- to 3-fold in the *in vitro* mutants and 1.4-fold in the *in vivo* evolved mutant CMY-33 (Table 2).

**Table 2:** Relative fold changes of transcription levels and IncI1 plasmid copy number in *Ec*-1 and its cefepime-resistant derivatives^a^.

Strain	Relative *ompF* transcript level ± SD	Relative *ompC* transcript level ± SD	Relative *bla*_CMY_ transcript level ± SD	Relative IncI1 plasmid copy number ± SD
*Ec*-1	1	1	1	1
EC-4	1.74 ± 0.54	1.09 ± 0.19	4.27 ± 0.2	2.27 ± 0.42
EC-16	0.91 ± 0.52	0.85 ± 0.06	12.76 ± 0.95	2.36 ± 0.24
EC-32	0.60 ± 0.09	0.53 ± 0.03	14.12 ± 0.37	2.79 ± 0.07
*Ec*-2	1.06 ± 0.31	0.54 ± 0.1	2.48 ± 0.04	1.4 ± 0.02

*SD, standard deviation. ^a^All transcript levels are relative to Ec-1.*

Transcript levels of *ompC* gradually decreased up to twofold in the *in vitro* and *in vivo* mutants compared to *Ec*-1. A similar pattern was observed in the transcript levels of *ompF* in the *in vitro* mutants, but not in the *in vivo* mutant strain *Ec*-2 (Table 2). Consistently, SDS PAGE analysis did not evidence any lack of OMPs in the resistant strains (Supplementary Figure S2).

### Growth Kinetics

**FIGURE 2:**
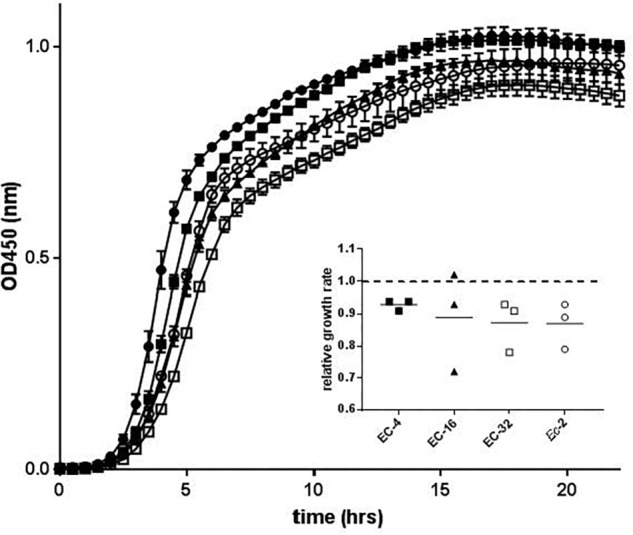
Growth kinetics of strain *Ec*-1 and its *in vitro* and *in vivo* mutant derivatives. The mean OD_450_ (±SEM) over time of three biological replicates is shown for *Ec*-1 (filled circles), EC-4 (filled squares), EC-16 (filled triangles), EC-32 (open squares), and *Ec*-2 (open circles). The inset shows the relative growth rate in minutes for each mutant strain compared to the parental strain.

We next evaluated the *in vitro* fitness of the mutant strains compared to *Ec*-1 in terms of growth under laboratory conditions. Plotting OD_450_ against time revealed that all resistant strains evolved from *Ec*-1 exhibited a slight growth defect compared to the parental strain (Figure 2). However, there was no statistically significant difference in the relative growth rates of the mutant strains compared to *Ec*-1 (Figure 2).

## Discussion

Reports of FEP resistance evolution in CMY-2-producing *E. coli* isolates are still rare. However, there has been a constant increase in the number of such strains isolated from different sources, such as humans, animals and food in Switzerland, as well as in other parts of the world (Liebana et al., 2013; Seiffert et al., 2013a,b). Furthermore, detection of AmpC production and differentiation from ESBL-phenotypes is problematic in diagnostic laboratories, or might not be sought after following a negative confirmatory test for ESBLs, which could potentially lead to underestimate the prevalence of AmpC-producers (Jacoby, 2009).

Switzerland is among the major consumers of FEP per capita in Europe (Pluss-Suard et al., 2012), which could favor the selection of resistant mutants. Low antibiotic levels at the site of infection due to suboptimal dosing or inadequate distribution in some body compartments and tissues represent a major driver in resistance evolution (Andersson and Hughes, 2014). In this regard, FEP concentrations in the lung tissues seem to reflect blood concentrations well (Breilh et al., 2001), but were also shown to be extremely variable in critically ill patients (Lipman et al., 1999). Of note, some patients in the latter study showed very low plasma levels of FEP, i.e., in a range that could potentially promote resistance evolution in *Pseudomonas aeruginosa* (Fantin et al., 1994). For this reason, alternative dosing regimens, such as continuous infusion, were suggested as valuable options to maintain sustained drug levels over time (Boselli et al., 2003).

In this study, we show that *in vitro* exposure of the *bla*_CMY–2_-expressing *Ec*-1 strain to sublethal concentrations of FEP leads to the selection of FEP-resistant mutants with similar resistance mechanisms as observed *in vivo*, i.e., mutations in *bla*_CMY_ and an increase in its transcript levels.

The evolution of CMY-2 to CMY-69 under selective pressure confirms the H-10 helix as a mutational hotspot in this class C β-lactamase in the development of FEP resistance. In fact, it has been previously shown that insertions, deletions, or substitutions in the H-10 helix of class C β-lactamases may result in the expansion of the substrate spectrum by an increased hydrolytic activity due to a better accommodation of cephalosporins, such as FEP, into the active site (Kim et al., 2006). It is interesting to note that evolution of the CMY-2 occurred *in vitro* through a SNP in the H-10 helix, while *in vivo* through a 6 bp deletion in the same region. The latter genetic event is less likely to occur and raises the question, whether a more radical modification of the enzyme’s active site is needed for the development of FEP resistance *in vivo*. Of note, *Ec*-2 exhibited a 10-fold higher MIC_FEP_ compared to EC-4, despite the lower *bla*_CMY_ mRNA levels. Deletions in the H-10 helix of the CMY-33 or CMY-44 variants were previously associated with reduced susceptibility to FEP in clinical *E. coli* isolates, with the MIC_FEP_ ranging from 6 to 96 mg/L among the tested CMY-33-producing strains (Doi et al., 2009). Nevertheless, the variability in the MIC_FEP_ also suggested that additional background-specific genetic factors may contribute to FEP resistance (Doi et al., 2009).

Overproduction of the beta-lactamase, as well as reduced drug permeation due to the loss or downregulation of porins, such as OmpF and OmpC, represent other common resistance mechanisms known to affect beta-lactam resistance in *E. coli*, and, notably, both mechanisms were recently linked to the development of carbapenem resistance in *bla*_CMY–2_-harboring *E. coli*, both in clinical isolates and *in-vitro* generated mutants (Goessens et al., 2013; van Boxtel et al., 2017). In contrast to this previous study, in which porin loss represented the first step in the evolution of meropenem resistance, only a slight downregulation of porins (mainly of OmpC), which was not detectable on SDS page analysis, was observed in the *in vivo* and *in vitro* mutants. This is consistent with previous observations suggesting that porin deficiency may have little impact in the development of FEP resistance in *E. coli* (Chen and Livermore, 1993). However, all mutants deriving from *Ec*-1 showed an increase in *bla*_CMY_ transcript levels. Particularly the *in vitro* mutants showed a gradual increase in *bla*_CMY_ mRNA compared to *Ec*-1 over time under constant selective pressure, which correlated with higher FEP MICs. Additionally, EC-16 and EC-32 developed resistance against piperacillin/tazobactam, while the parental strain and the other derivative mutants were susceptible. This phenomenon is likely attributable to the higher *bla*_CMY_ transcript levels in these two strains, consistent with a recent finding of a correlation between higher transcription levels of this gene and higher piperacillin/tazobactam MICs (Kurpiel and Hanson, 2012). However, while in this previous study the increase in *bla*_CMY_ mRNA perfectly correlated with the increase in plasmid copy number, in our study the 2- to 3-fold increase in plasmid copy number due to mutations in the *inc* antisense RNA gene could only partially account for the up to 14-fold increase in transcript levels. Based on our WGS data, we were unable to pinpoint any additional mutations, including in the promoter region of *bla*_CMY_, or any duplication or transposition events, which could explain this phenomenon. Thus, some transcriptional or posttranscriptional regulatory mechanisms may be involved in the observed increase of the *bla*_CMY_ mRNA levels over time, although we cannot completely exclude an unidentified genetic factor.

Interestingly, we identified in the *in vivo* mutant *Ec*-2 two mutations in *rpoC* which may represent compensatory mutations that could broadly affect gene transcription, and explain the lower *bla*_CMY–2_ transcript levels compared to the *in vitro* deriving strains. However, these mutations may also be directly associated with FEP resistance. For instance, a mutation in *rpoC* was previously shown to be sufficient, in absence of any additional resistance determinants, to induce cephalosporin resistance in *Bacillus subtilis* (Lee et al., 2013) due to an increase of the expression of alternative σ factors, which control the transcription of genes in response to cell envelope stress.

Efflux did not seem to play a major role in the FEP resistance of the *Ec*-1 derivatives, since MICs in the presence of PAbN, a broad-spectrum inhibitor of RND pumps in Gram-negative bacteria (Fernandez and Hancock, 2012), remained unaltered.

All mutants only exhibited a slight growth defect and no difference in terms of relative growth rate compared to the parental strain, indicating that, at least during growth in rich medium under laboratory conditions, there was no considerable fitness cost associated with the resistant phenotype. These *in vitro* conditions do not mimic the environment encountered by the bacteria in the host, however, the fact that *Ec*-2 evolved from *Ec*-1 *in vivo*, highlights the possibility that such strains may be able to successfully adapt and survive in a particular niche, such as in the lung or in abscesses, under selective pressure.

In conclusion, our study shows that mutations in *bla*_CMY_ and increase of its transcript levels are common resistance mechanisms involved in the development of FEP resistance under selective pressure both *in vitro* and *in vivo*. We acknowledge that further studies are needed to fully characterize the observed mechanisms of resistance and their contribution in the development of FEP resistance. However, our observations advocate for the importance to detect and differentiate AmpC and ESBL production in diagnostic laboratories and indicate that clinicians should be aware of the potential development of FEP resistance in CMY-2-producing *E. coli* isolates, most probably as a consequence of suboptimal drug concentrations during FEP treatment. We showed that exposure to sub-lethal concentrations of FEP resulted in a continuous increase in the MIC_FEP_, suggesting that resistance to this drug in CMY-2-producing strains may increase over time just as a consequence of suboptimal drug concentrations. Therefore, optimized dosing strategies, e.g., by shorter dosing intervals or continuous infusion of FEP led by therapeutic drug monitoring, may be considered as valuable options for maintaining adequate drug levels and avoid resistance evolution.

## Data Availability

The datasets generated for this study can be found in NCBI, GenBank, *Ec*-1,QEVU00000000; EC-4, QDKR00000000; EC-16, QDKS00000000; EC-32, QDKT00000000; and *Ec*-2, QDKU00000000.

## Author Contributions

VD wrote the manuscript, helped to conducting the study, and did part of the molecular analysis. MS did part of the laboratory work and helped to writing the manuscript. JP was involved in data analysis. AA advised on the statistical part. HF helped to conceiving the study. BBF conceived the study, did the laboratory work and analysis of data, and helped to writing the manuscript. All authors critically revised the manuscript.

## Conflict of Interest Statement

The authors declare that the research was conducted in the absence of any commercial or financial relationships that could be construed as a potential conflict of interest.

## Footnotes

1 https://cge.cbs.dtu.dk/services/

2 http://www.geneious.com
