# Supplementary material for: Gradual in vitro Evolution of Cefepime Resistance in an ST131 Escherichia coli Strain Expressing a Plasmid-Encoded CMY-2 β-Lactamase
Source: Front Microbiol. 2019 Jun 12;10:1311. doi: 10.3389/fmicb.2019.01311 (PMC6581752; doi:10.3389/fmicb.2019.01311)
Supplement: Supplementary file 1 [file Data_Sheet_1.PDF]

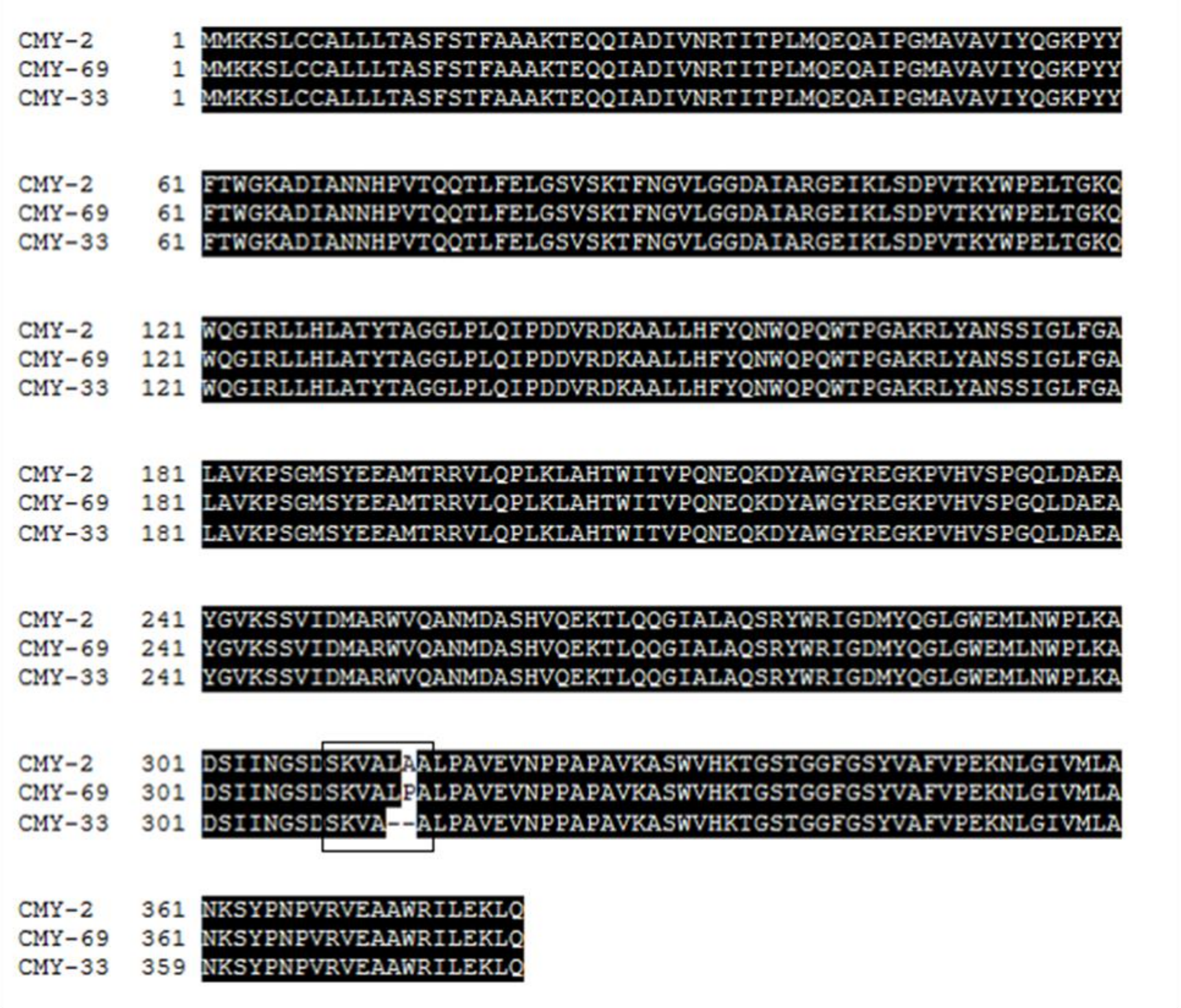

Figure S1. Amino acid sequence alignment of CMY-2, CMY-69 and CMY-33.

The H-10 helix is highlighted in the box

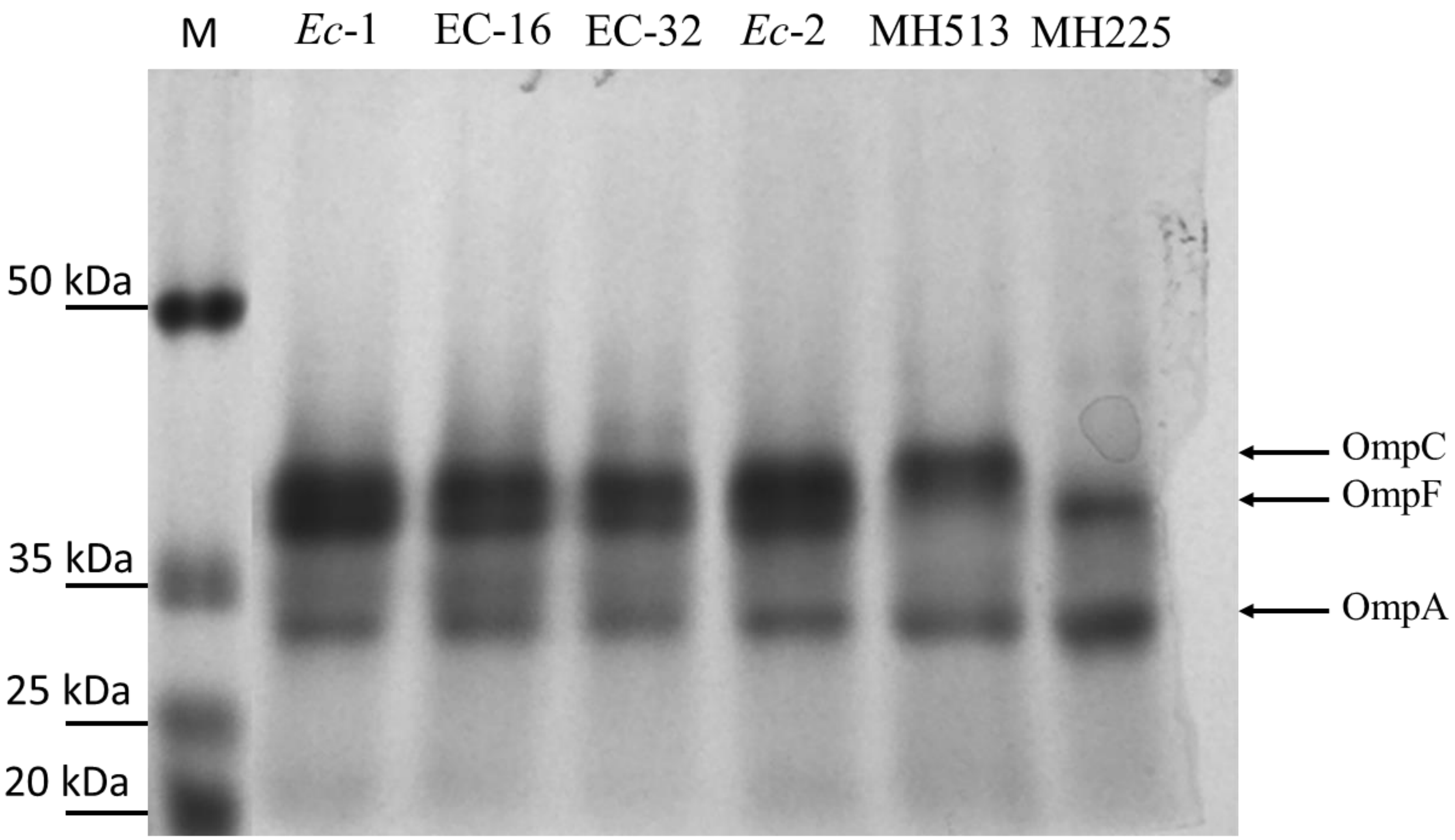

Figure S2. SDS PAGE of outer membrane proteins of strain *Ec*-1 and its derivative mutants. M, Marker

Table S1. Primers used in this study

Gene/primer names	Sequence 5’ → 3’	Use	Reference
IncI1-F<br>IncI1-R	CTGTTATTAGCTGTGCAGAGG<br>CTTCTGACTTACTGCCCGTAC	IncI1 identification and real-time plasmid copy number assays	(1)
ampD F RT<br>ampD R RT	CCTCACCACATTACGATTGC<br>CCTGCGGATCAATAGTTCC	*E. coli ampD* real-time gene copy number endogenous control primers	(1)
CMY-F<br>CMY-R	GGG CCC GGA CAC CYT TTT GC<br>GGG CCC GGA CAC CYT TTT GC	Whole gene amplification & sequencing	(2)
CMY_RT F<br>CMY_RT R	CCAGCATTGGTCTGTTTGGC<br>TCCAGGTATGCGCCAGTTTT	Real-time gene expression analysis	*This study*
OmpF F<br>OmpF R	GTTCGCTGCCAGGTAGATGT<br>CGGTGTTGGCGGTTCTATCA	Real-time gene expression analysis	*This study*
OmpC F<br>OmpC R	AGTTAAAGTACTGTCCCTCCTGG<br>AGAAATAGTGCAGGCCGTCT	Real-time gene expression analysis	*This study*
rpoD F<br>rpoD R	CAGGTTCAATGCTCCGTTCG<br>TGGGAAAGCTCAGAACCGAC	Real-time gene expression analysis endogenous control primers	(3, 4)

References

1. **Kurpiel PM, Hanson ND.** 2012. Point mutations in the inc antisense RNA gene are associated with increased plasmid copy number, expression of *bla*CMY-2 and resistance to piperacillin/tazobactam in *Escherichia coli*. J Antimicrob Chemother **67:**339-345.
2. **D'Andrea MM, Nucleo E, Luzzaro F, Giani T, Migliavacca R, Vailati F, Kroumova V, Pagani L, Rossolini GM.** 2006. CMY-16, a novel acquired AmpC-type beta-lactamase of the CMY/LAT lineage in multifocal monophyletic isolates of *Proteus mirabilis* from northern Italy. Antimicrob Agents Chemother **50:**618-624.
3. **Sauer U, Canonaco F, Heri S, Perrenoud A, Fischer E.** 2004. The soluble and membrane-bound transhydrogenases UdhA and PntAB have divergent functions in NADPH metabolism of *Escherichia coli*. J Biol Chem **279:**6613-6619.
4. **Marcoleta AE, Berrios-Pasten C, Nunez G, Monasterio O, Lagos R.** 2016. *Klebsiella pneumoniae* Asparagine tDNAs Are Integration Hotspots for Different Genomic Islands Encoding Microcin E492 Production Determinants and Other Putative Virulence Factors Present in Hypervirulent Strains. Front Microbiol **7:**849.
